# Rodent-associated *Bartonella* Febrile Illness, Southwestern United States

**DOI:** 10.3201/eid1207.040397

**Published:** 2006-07

**Authors:** Jonathan Iralu, Ying Bai, Larry Crook, Bruce Tempest, Gary Simpson, Taylor McKenzie, Frederick Koster

**Affiliations:** * US Public Health Service, Gallup, New Mexico, USA;; † University of Colorado, Boulder, Colorado, USA;; ‡ New Mexico Department of Health, Santa Fe, New Mexico, USA;; § The Navajo Nation, Window Rock, Arizona, USA;; ¶ University of New Mexico Health Science Center, Albuquerque, New Mexico, USA;; # Lovelace Respiratory Research Institute, Albuquerque, New Mexico, USA

**Keywords:** *Bartonella*, liver function tests, hepatic injury, leukopenia, thrombocytopenia, fever, zoonosis, research

## Abstract

Serum specimens from 114 patients hospitalized with a febrile illness were tested with an indirect immunofluorescence assay (IFA) using *Bartonella* antigens prepared from 6 species of sigmodontine rodents and 3 known human *Bartonella* pathogens: *B. henselae*, *B. quintana*, and *B. elizabethae*. Acute- and convalescent-phase serum samples from 5 of these patients showed seroconversion with an IFA titer >512 to rodent-associated *Bartonella* antigens. The highest titer was against antigen derived from the white-throated woodrat (*Neotoma albigula*), although this rodent is not necessarily implicated as the source of infection. Three of the 5 who seroconverted showed no cross-reaction to the 3 *Bartonella* human pathogens. Common clinical characteristics were fever, chills, myalgias, leukopenia, thrombocytopenia, and transaminasemia. Although antibodies to *Bartonella* are cross-reactive, high-titer seroconversions to rodent-associated *Bartonella* antigens in adults with common clinical characteristics should stimulate the search for additional *Bartonella* human pathogens.

## Abstract 2

Patients showed seroconversion to rodent-associated *Bartonella* antigens, but not to *Bartonella* pathogenic for humans.

The discovery of hantavirus pulmonary syndrome and its high death rate in the southwestern United States resulted in greater vigilance in evaluating patients with acute febrile illness, particularly those with thrombocytopenia (*1*). Clinicians soon became aware of substantial numbers of hospitalized patients with a severe flulike prodrome and thrombocytopenia. In spite of conventional culture and serologic analysis for known pathogens and diseases, including hantaviruses, plague, tularemia, relapsing fever, spotted fever, murine typhus, and Q fever, no diagnosis could be made. To assist physicians in identifying treatable pathogens, we submitted serum to reference laboratories for diagnostic seroassays directed at known pathogens and organisms not previously associated with human disease. A concept of the role of rodent-associated bartonellae as a cause of unexplained febrile illness in the western United States has been recently developed (M. Kosoy, pers. comm.). We considered the possibility that some cases in our study were caused by *Bartonella* species.

Among at least 20 known species and subspecies of *Bartonella*, 5 have been identified as causes of human disease in North America (*2**,**3*). *B*. *henselae* causes cat-scratch disease with regional lymphadenitis and occasionally hepatosplenic disease in the immunocompetent host, and bacillary angiomatosis, cerebritis, or peliosis hepatis in the immunocompromised host (*4**–**6*). Louseborne *B*. *quintana* causes trench fever, aseptic meningitis, bacteremia, endocarditis, or bacillary angiomatosis (*4**,**7**–**9*). Recently isolated cases of infection with *B*. *elizabethae* (*10*), *B*. *vinsonii* subsp. *arupensis* (*11*), and *B*. *washoensis* (*12*) suggest that the spectrum of *Bartonella* infections may continue to expand.

Many mammals, including numerous species of rodents, are commensally infected with *Bartonella* species in North America (*12**–**15*). We sought serologic evidence for human bartonellae infection in serious febrile illnesses in the Four Corners region, using diverse *Bartonella* antigens in an indirect immunofluorescence assay (IFA) (*13*). We report 7 years' cumulative experience in diagnostic referrals, including 5 cases showing seroconversion, and 4 cases with a single high titer, to *Bartonella* antigens derived from strains isolated from rodents, particularly the white-throated woodrat (*Neotoma albigula*) captured in New Mexico.

## Materials and Methods

### Patients

From July 1993 to June 2001, 114 patients 15–78 years of age were referred by their physicians for assistance in diagnosing a febrile illness with a duration <12 days at the time of admission. One hundred patients were hospitalized in New Mexico, 10 in Arizona, and 4 in Colorado. All patients were hospitalized on the basis of the attending physician's decision concerning severity of illness, the possibility of hantavirus infection in the prodrome phase, and the need for diagnostic studies, supportive care, and presumptive antimicrobial-drug therapy. At the time specimens were collected, results of conventional microbiologic assays and diagnostic serologic analysis were negative or unavailable.

Patients were divided into 4 clinical groups according to conventional diagnostic results (Table 1). Seventy-six patients (group A) had an acute undifferentiated febrile illness without pulmonary, cardiac, or renal manifestations. Twelve patients (group B) had bacterial lobar pneumonia (11 patients) or acute respiratory distress syndrome (1 patient) diagnosed by typical signs and symptoms, hypoxemia, pulmonary infiltrates, and prompt clinical response to β-lactam antimicrobial drugs (*16**,**17*). Twelve patients (group C) had hantavirus cardiopulmonary syndrome diagnosed by strip immunoblot serology (*18*) and reverse transcription–polymerase chain reaction (RT-PCR) of serum (*19*). Fourteen patients (group D) had an acute febrile syndrome without pulmonary manifestations and with a diagnosis established by conventional blood culture, serology, or PCR; this group included 3 patients with *Escherichia coli* sepsis, 2 with *E*. *coli* pyelonephritis, 3 with Rocky Mountain spotted fever, 1 with acute *Staphylococcus aureus* aortic valve endocarditis, 1 with bubonic plague, 1 with acute Q fever, 1 with parvovirus infection, 1 with acute rheumatic fever, and 1 with acute lupus erythematosis. All patients (except those in group D) had at least 2 negative blood cultures, negative spinal fluid cultures and cytometrics when appropriate, negative hantavirus serologic results (except group C), and negative serologic results for plague, tularemia, Q fever, spotted fever, and *Ehrlichia* species ordered at the discretion of the attending physician. Except for hypertension (5 patients) and chronic alcoholism (12 patients), no patient had underlying disease such as diabetes, malignancy, or HIV infection. The charts were reviewed retrospectively by the investigators. The study was approved by the institutional review boards of the University of New Mexico and the Navajo Nation.

**Table 1:** Rodent-associated *Bartonella* serologic results in 114 adults with acute febrile illness, southwestern United States

Clinical diagnosis	Total	No. thrombocytopenic	No. leukopenic	Titer to *Neotoma albigula*–associated *Bartonella* antigen				
				<64	128	256	512	>1,024
Undifferentiated fever	76	55	43	52	11	2	5	6
Bacterial pneumonia	12	4	4	12	0	0	0	0
Hantavirus pulmonary syndrome	12	12	0	11	1	0	0	0
Other febrile illnesses	14	9	4	11	0	1	0	2

### Serologic Analysis

Citrated and clotted blood was collected within 24 hours of admission from 90 patients (acute-phase sample), 7–42 days after admission from 10 patients, and at admission and during convalescence from 14 patients (all in group A). Plasma was immediately frozen at –80°C. An IFA was performed as previously described (*13*). All antigens were prepared at the Bacterial Zoonoses Branch, Centers for Disease Control and Prevention (CDC), Fort Collins, Colorado.

Vero E6 monolayers were infected separately with 1 of 9 strains of *Bartonella*: 3 strains (*B*. *quintana, B*. *henselae*, and *B*. *elizabethae*) were isolated from humans and 6 strains were isolated from the meadow vole (*Microtus pennsylvanicus*), white-throated woodrat (*N*. *albigula*), deer mouse (*Peromyscus maniculatus*), cotton rat (*Sigmodon hispidus*), Ord kangaroo rat (*Dipodomys ordi*), and rock squirrel (*Spermophilus variegatus*). Plasma was diluted 1:32 in phosphate-buffered saline, placed in antigen-containing wells, incubated at 37°C for 30 minutes, washed, and incubated at 37°C for 30 minutes with rabbit antihuman immunoglobulin (Ig) conjugated with fluorescein isothiocyanate. Positive samples were then tested in serial 2-fold dilutions on monolayers infected with 1 of 9 *Bartonella* strains. Mouse hyperimmune sera were produced by injection of BALB/c mice with the same *Bartonella* strains that were used for the antigen preparations. These sera were used as IFA-positive controls (titers >1,000 in each assay). Results were tabulated without knowledge of the patient's clinical status.

## Results

Serum samples from 114 patients with acute febrile illness, including 14 with both acute- and convalescent-phase serum samples, were tested at a dilution of 1:32 by IFA with a panel of 9 *Bartonella* antigens. All positive samples were retested at a dilution of 1:32 and at doubling dilutions to 1:4,096. In 12 of 13 cases with titers <512 to any rodent-associated antigen, the titer to the *N*. *albigula*–associated *Bartonella* antigens (NA-AB antigens) were the highest measured. Therefore, only the titers to NA-AB antigens are shown in Table 1. IFA titers to NA-AB >128 were observed more often in undifferentiated febrile illness (group A, 24 of 76) than in the 3 groups with specific diagnoses (groups B–D, 4 of 38) (χ^2^ = 4.98, p = 0.026, using Yates' correction). Among 24 patients in group A with titers >128, a total of 11 had convalescent-phase titers >512. Clinical information was sufficient to analyze for 9 of these 11 patients: 5 patients with both acute- and convalescent-phase titers (Table 2) and 4 patients with only a convalescent-phase titer (Table 3). Nine patients in group A with both acute- and convalescent-phase serum samples showed no increase in titer or a titer >64.

**Table 2:** Clinical and laboratory data of 5 adults with undifferentiated fever and seroconversion to *Neotoma albigula*–derived *Bartonella* antigens*

Patient no., age (y), sex	DOI	T (°C)†	Leukocytes × 10^3^/μL‡	PLT × 10^3^/μL‡	HCT (%)†	AST (U/L)†	BIL (mg/dL)†	LDH (U/L)†	Doubling dilution end titer (acute/convalescent phases)§				
									*Bartonella* from *Neotoma*	*B*. *vinsonii from Microtus pennsylvanicus*	*B*. *quintana*	*B*. *henselae*	*B*. *elizabethae*
1, 55, F	5	39.7	2.7	147	44	183	1.5	167	256/4,096	64/64	<32/64	<32/<32	64/64
2, 30, M	5	39.3	3.2	110	50	85	1.5	206	256/1,024	128/1,024	64/512	64/256	64/512
3, 34, F	6	39.7	3.5	95	44	324	1.7	190	<32/1,024	<32/256	<32/64	64/256	<32/64
4, 29, M	2	39.2	17.9	226	48	ND	ND	ND	<32/512	32/64	<32/<32	<32/<32	32/32
5, 23, F	2	38.8	5.0	125	40	ND	ND	130	32/512	<32/64	<32/<32	<32/128	<32/64

*DOI, day of symptomatic illness at hospitalization; T, temperature; PLT, platelet count; HCT, hematocrit ; AST, aspartate aminotransferase; BIL, bilirubin; LDH, lactate dehydrogenase; ND, not determined. †Highest value during 2–6 d of hospitalization. ‡Minimum value. §Convalescent-phase titers 2–6 wk after hospital admission.

**Table 3:** Clinical and laboratory data of 4 adults with undifferentiated fever and a single convalescent-phase titer to *Neotoma albigula*–derived *Bartonella* antigens*

Patient no., age (y), sex	DOI	T (°C)†	Leukocyte × 10^3^/μL‡	PLT × 10^3^/μL‡	HCT (%)†	AST (U/L)†	BIL (mg/dL)†	LDH (U/L)†	Doubling dilution end titer (convalescent phase)§				
									*Bartonella* from *Neotoma*	*B. vinsonii* from *Microtus pennsylvanicus*	*B. quintana*	*B. henselae*	*B. elizabethae*
6, 42, M	2	39.1	23.3	11	47	4,580	4.3	16,000	2,048	64	<32	<64	128
7, 17, M	3	38.8	3.3	108	43	60	1.4	229	1,024	256	256	64	512
8, 23, F	3	39.0	3.9	245	44	834	5.2	NA	512	256	256	64	64
9, 32, M	7	39.0	2.9	35	44	1,049	3.8	1,248	512	NA	NA	NA	NA

*DOI, day of symptomatic illness at hospitalization; T, temperature; PLT, platelet count; HCT, hematocrit; AST, aspartate aminotransferase; BIL, bilirubin; LDH, lactate dehydrogenase; NA, not available. †Highest value during 2–6 d of hospitalization. ‡Minimum value. §Convalescent-phase titers 2–6 wk after hospital admission.

Of 24 patients with pneumonic disease (groups B and C), only 1 had a titer of 128 to NA-AB antigens. Of 14 patients with other diagnosed febrile illnesses (group D) not listed in Table 2 and Table 3, three had high titers to NA-AB antigens (Table 1). A 35-year-old man with aortic valve endocarditis and cultures of blood and valve positive for *S*. *aureus* had an NA-AB titer of 1,024 on admission and the following day. A 30-year-old man with fever, myalgias, headache, thrombocytopenia, and leukopenia with admission serum positive by PCR for *Borrelia hermsii* (tickborne relapsing fever) had an acute-phase (day 1) titer of 256 and a convalescent-phase (day 24) titer of 1,024 to NA-AB antigens. A 23-year-old woman with fever and acute hepatic injury had positive convalescent-phase (day 28) IgM phase I (512) and IgG phase II (1,024) titers for *Coxiella burnetii* antigens and an NA-AB antigen titer of 256 in a convalescent-phase serum sample.

Five of the 14 patients with acute- and convalescent-phase serum samples in group A showed a >4-fold increase in titer to NA-AB antigens and convalescent-phase titers >512 on days 14, 7, 7, 12, and 42, respectively, after admission (Table 2). Each of the 5 who seroconverted had a clinical syndrome characterized by fever (temperature >39°C), chills, pronounced myalgias in the back and thighs, nausea, and headache. Two who seroconverted had a sore throat and 2 had diarrhea, but none had other upper or lower respiratory symptoms, abnormal chest radiograph results, lymphadenopathy, hepatosplenomegaly, bleeding, rash, altered consciousness, or abnormal neurologic findings. Thrombocytopenia and leukopenia were common (Table 2 and Table 3), but no patients had evidence of coagulopathy, or cardiac, pulmonary, renal, or neurologic disease.

Four other patients in group A had a single titer >512 to NA-AB antigens on days 21, 7, 20, and 23, respectively, after admission (Table 3). This group had elevated levels of serum transaminase, bilirubin, and alkaline phosphatase, which is indicative of active hepatitis. These 4 patients were treated with doxycycline, and all recovered without sequelae. Of the 9 patients listed in Tables 2 and 3, one had a diagnosis of chronic alcoholism (patient 6, Table 3). All 9 were negative for hepatitis A, B, and C; Q fever; Rocky Mountain spotted fever; murine typhus; leptospirosis; granulocytic or monocytic ehrlichiosis; plague; and tularemia; they also had negative titers for HIV, hantavirus, and antinuclear antibody. Patients 6, 8, and 9 were tested for antibody to hepatitis E at the Hepatitis Branch of CDC in Atlanta, Georgia, and were negative (M. Favorov, pers. comm.). Patients 1, 4, and 6 had 6-, 3-, and 3-fold lower titers, respectively, to the known *Bartonella* pathogen antigens compared with the titer to NA-AB antigens (Table 2 and Table 3).

## Discussion

This study provides preliminary serologic evidence for a *Bartonella* or *Bartonella* cross-reactive species that is causing acute febrile illness in immunocompetent adults in the rural southwestern United States. Five patients who seroconverted to rodent-associated antigen had fever, myalgias, headache, and chills with varying degrees of leukopenia, mild hepatitis, and thrombocytopenia. Four other patients with a single elevated titer 2–5 weeks into their illness had more severe hepatic injury. In the absence of culture- or PCR-positive evidence of *Bartonella* infection in any of these patients, the interpretation of these serologic observations is related to the cross-reactivity between *Bartonella* species as well as non-*Bartonella* species, interpretation of the quantitative IFA titer, variations among pathogens to stimulate antibody responses, timing of serum specimen collection, and the route of exposure.

Although antigens derived from *Bartonella* isolated from *N*. *albigula* were used, this process does not imply that the human infection was caused by a *Bartonella* strain that naturally infects *N*. *albigula*. Serologic cross-reactions among *Bartonella* species are common (*20*), and the IFA is unable to distinguish between infection with *B*. *quintana* or *B*. *henselae* (*21*). The cross-reactivity between rodent-associated and known *Bartonella* pathogen–associated antigens was expected and found to some degree in nearly all cases. We did not find clear evidence for infection with *Bartonella* species known to cause disease in humans, including *B*. *henselae*, *B*. *quintana*, *B*. *vinsonii*, and *B*. *elizabethae*, in the sense that titers to rodent-associated, particularly NA-AB, antigens were always higher than those for known human *Bartonella* species. The lack of cross-reactivity in 3 patients is consistent with a rodent-associated *Bartonella* infection, although infection with a *Bartonella* associated with a nonrodent animal cannot be ruled out (*22*).

Identification of *Bartonella* infections in humans in the southwestern United States is important because cat-scratch disease is not common in this region, and cat fleas, presumed vectors for *B*. *henselae*, do not naturally exist in such arid environments (*23*). Cross-reactivity between *Bartonella* antigens and antigens of *C*. *burnetii* and *Chlamydia* species has been demonstrated (*24**,**25*). Except for the woman in group D who had clear evidence of acute Q fever hepatitis, significant *Bartonella* titers >128 were not associated with detectable antibody to phase I or II *Coxiella* antigens in the complement fixation test in all 8 patients tested. None of the patients had a condition associated with nonspecific immune stimulation such as HIV infection, injection drug use, or collagen vascular disease that could account for false-positive results.

The IFA was developed at CDC (*21*) and has been assessed most extensively in the diagnosis of *B*. *henselae* and *B*. *quintana* infection in the United States (*20*). At the National Referral Center of CDC, a titer of 64 is considered positive (*20*). When a strict case definition is used for cat-scratch disease, this titer has a sensitivity of ≈80% and a specificity of 93% to 96% (*20**,**21**,**26*). Other investigators have found greater specificity when titers of 128 (*27*), 256 (*25*), or 512 (*28*) were used to diagnose cat-scratch disease. An IFA titer of 512 to *B*. *henselae* in adults with no exposure to cats or illness compatible with cat-scratch disease was uncommon (<1%) in 1 study in Germany (*27*). We used a conservative threshold IFA titer of 512 to present clinical data on 9 patients based in part on this experience with cat-scratch disease, recognizing that immunogenicity to immunodominant antigens may vary among species of the same genus. The usefulness of a single titer of 1:512 to NA-AB antigens (Table 3) is unknown because IFA titers to *B*. *henselae* persist during the first year after infection (*20*).

The clinical syndrome associated with seroconversion to NA-AB antigens was characterized by either a brief undifferentiated febrile illness or fever accompanied by hepatic injury. Clinical evidence for inflammation in the lung, heart, kidney, and nervous system was not apparent. Infection with *B*. *henselae*, particularly in immunocompromised hosts, has been documented to involve the liver (*2*). Moreover, thrombocytopenia and leukopenia, which were common in our small sample of febrile patients, have also been associated with *B*. *quintana* infection (*29*) in immunocompetent adults and with *B*. *henselae* infection in immunocompromised adults (*2*). No patient had intraerythrocytic bacilli visible on Giemsa-stained blood smear (*30*) (F. Koster, unpub. data). A clear definition of the syndrome awaits definitive identification based on culture of the pathogenic species from patients. Thus, a concerted effort to identify acute infections with rodent-associated *Bartonella* should be undertaken with specific serologic assays as well as intensive PCR-based diagnostics and culture techniques specific to the fastidious *Bartonella* genus.
